# Risk Factors of Rhino Orbital Mucormycosis

**DOI:** 10.7759/cureus.33145

**Published:** 2022-12-30

**Authors:** Yugandhara Patade, Rashmi G

**Affiliations:** 1 Ophthalmology, Sri Devaraj Urs Academy of Higher Education and Research, Kolar, IND

**Keywords:** pandemic, diabetes mellitus, covid 19, corticosteroids, mucormycosis

## Abstract

Purpose: To determine the clinical presentation and risk factors associated with rhino orbital mucormycosis.

Introduction: Mucormycosis is a rapidly progressive fungal infection caused by filamentous fungi in the Mucoraceae family. In large numbers, they release spores into the air, and humans get exposed through inhalation. The spores inoculate in the paranasal sinuses and nasopharynx and subsequently spread to the orbit and intracranial cavity. The present COVID-19 pandemic has witnessed a resurgence of rhino-orbital mucormycosis cases, mainly seen in patients with immunocompromised status. Hence, our study evaluated the risk factors and clinical features of rhino orbital mucormycosis.

Methods: This was a prospective, single-center, cross-sectional study. Patients attending tertiary care centers fulfilling the inclusion criteria were evaluated with a detailed history including sociodemographic profile, occupation, history of fever, COVID-19 infection, steroid or immunosuppressant use, organ transplants, diabetes mellitus and use of oxygen (O2). A complete ophthalmic evaluation was performed, including best-corrected visual acuity, anterior segment evaluation with slit lamp biomicroscopy, and posterior segment evaluation using indirect ophthalmoscopy. Computed Tomography (CT) scan of paranasal sinuses was done for all the patients, and Magnetic Resonance Imaging (MRI) for a few patients if indicated. Intraoperatively, samples were sent for Potassium Hydroxide (KOH) stain while debridement and patients with positive results were included in the study.

Results: Forty participants were included, out of which 34 (85%) were males and six (15%) were females. The mean age of the patients was 51.75 years. Out of 40 patients, 29 (72.5%) had h/o COVID-19 infection, 30 (75%) were known type 2 diabetes mellitus, 25 (62.5%) had a h/o steroid intake and 25 (62.5%) had a history of O2 use. 17 (42.5%) patients presented with low vision, out of which 15 had no light perception. 30 (75%) patients had ptosis, 22 (55%) patients presented with proptosis, 15 (37.5%) patients had limited ocular motility, 11 (27.5%) had complete ophthalmoplegia, and 11 (27.5%) patients had central retinal artery occlusion.

Conclusion: Rhino orbital Mucormycosis is more prevalent in patients with COVID-19 infection, especially those who have used steroids and oxygen and with type 2 diabetes mellitus. Early presentation with treatment can prevent further ocular morbidity.

## Introduction

Mucormycosis is a rapidly progressive fungal infection caused by filamentous fungi in the mucoraceae family. They are ubiquitous, mainly in soil, and occur naturally in the environment, body surfaces, and orifices [1,2]. These fungi develop rapidly, releasing large numbers of spores into the air to which human beings are often exposed through inhalation [1,2]. The spores inoculate in the paranasal sinuses and the nasopharynx. Subsequently, the spread to orbit and intracranial cavity in people with decreased immunity is seen [1,2]. These fungal hyphae are angioinvasive, causing necrotizing vacuities and thrombosis, resulting in tissue infarcts and necrosis [3]. The COVID-19 infection caused by the novel severe acute respiratory syndrome coronavirus 2 (SARS-CoV-2) may range from mild disease to life-threatening conditions, including Mucormycosis, and it has been observed to be one of the most common associations recently [4]. The pandemic of COVID-19 has witnessed a surge in cases of rhino-orbital Mucormycosis [1]. In the last few years, diabetes mellitus and renal diseases have been the common predisposing factors for mucormycosis [5]. Extensive use of corticosteroids, broad-spectrum antibiotics, and monoclonal antibodies may lead to the development of fungal disease or its exacerbation [4]. The most common underlying illnesses of rhino orbital cerebral mucormycosis are diabetes mellitus, hematological malignancies, hematopoietic stem cell transplantation, and solid organ transplantation [6]. Early diagnosis with the initiation of appropriate therapy can save both sight and life [1]. This study is aimed to assess the risk factors and presentation of patients with rhino orbital mucormycosis.

## Materials and methods

This was a prospective, single-center, cross-sectional study conducted at a tertiary care center from June 2021 to July 2022 during the second wave of the COVID-19 pandemic. The predominant strain was delta variant B.1.617. All the patients with microscopically confirmed mucormycosis were included in the study. The samples were obtained through nasal tissue biopsy and stained with potassium hydroxide (KOH). A total of 40 patients diagnosed with rhino orbital mucormycosis in the age group of 18 to 80 years were enrolled, and patients with bacterial infections were excluded from the study. Informed consent was obtained from all the patients included in the study, and Institutional Ethics committee approval was obtained. All the patients were evaluated with a detailed history including sociodemographic profile, occupation, COVID-19 infection, steroid use for more than three months or less than three months, use of immunosuppressants, any organ transplants, any chronic disease or disorder, use of oxygen mask for any health condition, history of prolonged hospital stay, recent or past history of surgeries. A complete ophthalmic evaluation was done, including best-corrected visual acuity, extraocular motility, pupillary reaction, slit lamp biomicroscopy for anterior segment evaluation, and posterior segment evaluation using indirect ophthalmoscopy. Patients underwent blood investigations, including complete blood count, erythrocyte sedimentation rate, C reactive protein, renal function test, liver function test, random blood sugar, D-dimer levels, and serum electrolytes. CT scan of paranasal sinuses, orbit, and brain was done for all the cases and MRI for a few patients as per indications. Mucormycosis was diagnosed based on the demonstration of broad aseptate hyphae with right-angled branching on 20% Potassium hydroxide (KOH) obtained from nasal cavity or paranasal sinuses specimens. All the patients received intravenous liposomal amphotericin B with a dosage of 3-5mg/kg body weight per day. Patients with sinus involvement were taken up for nasal endoscopic debridement, and specimens were sent for microbiology and histopathology by the department of otorhinolaryngology. Orbital debridement and exenteration were considered in patients with necrosis of orbital tissues without a perception of light. 

Statistical analysis

Data were entered into a Microsoft Excel data sheet and analyzed using SPSS 22 version software (IBM SPSS Statistics, Somers, NY, USA). Mean-qualitative or categorical data by frequency and percentage presented all quantitative measures.

## Results

The study included a total of 40 patients diagnosed with rhino orbital mucormycosis. The mean age of the patients was 51.75 years. Out of 40 patients, 34 (85%) were males, and six (15%) were females (Table 1). Out of 40 patients, 29 (72.5%) patients were COVID-19 positive, 30 (75%) patients were associated with type II diabetes mellitus, 25 (62.5%) patients had received corticosteroids, and 25 (62.5%) had a history of usage (Table 2). Seventeen (42.5%) patients presented with low vision, of which 15 had no perception of light (PL) (Table 3).

**Table 1:** Demographic profile of patients with rhino orbital mucormycosis

Gender	Males (%)	Females (%)
Total	34 (85)	06 (15)

**Table 2:** Risk factors of rhino orbital mucormycosis among diagnosed patients

Risk factors	Number of patients (percentage)
COVID-19 infection	29 (72.5)
Diabetes mellitus	30 (75)
Oxygen usage	25 (62.5)
Corticosteroids treatment	25 (62.5)

**Table 3:** Visual acuity in all the patients diagnosed with rhino orbital mucormycosis PL: perception of light

Visual acuity	Number (percentage)
6/6 to 6/18	8 (20)
<6/18 to 6/60	8 (20)
<6/60 to 3/60	7 (17.5)
<3/60 to Perception of Light (PL) +	2 (5)
No PL	15 (37.5)

**Figure 1:**
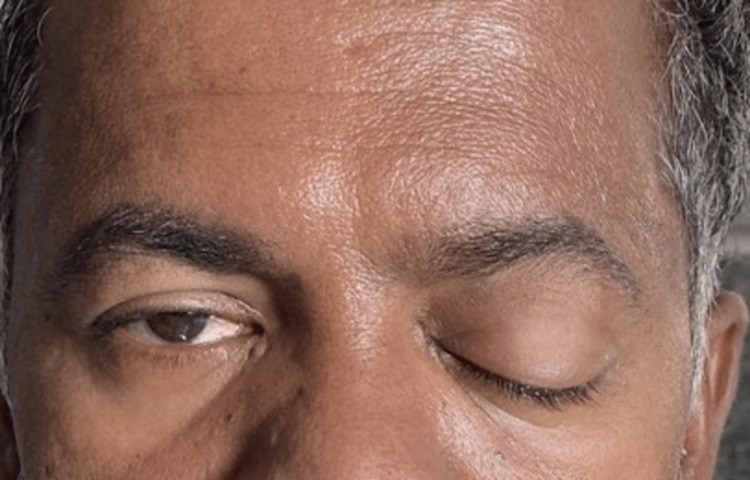
Patient showing complete unilateral ptosis.

**Figure 2:**
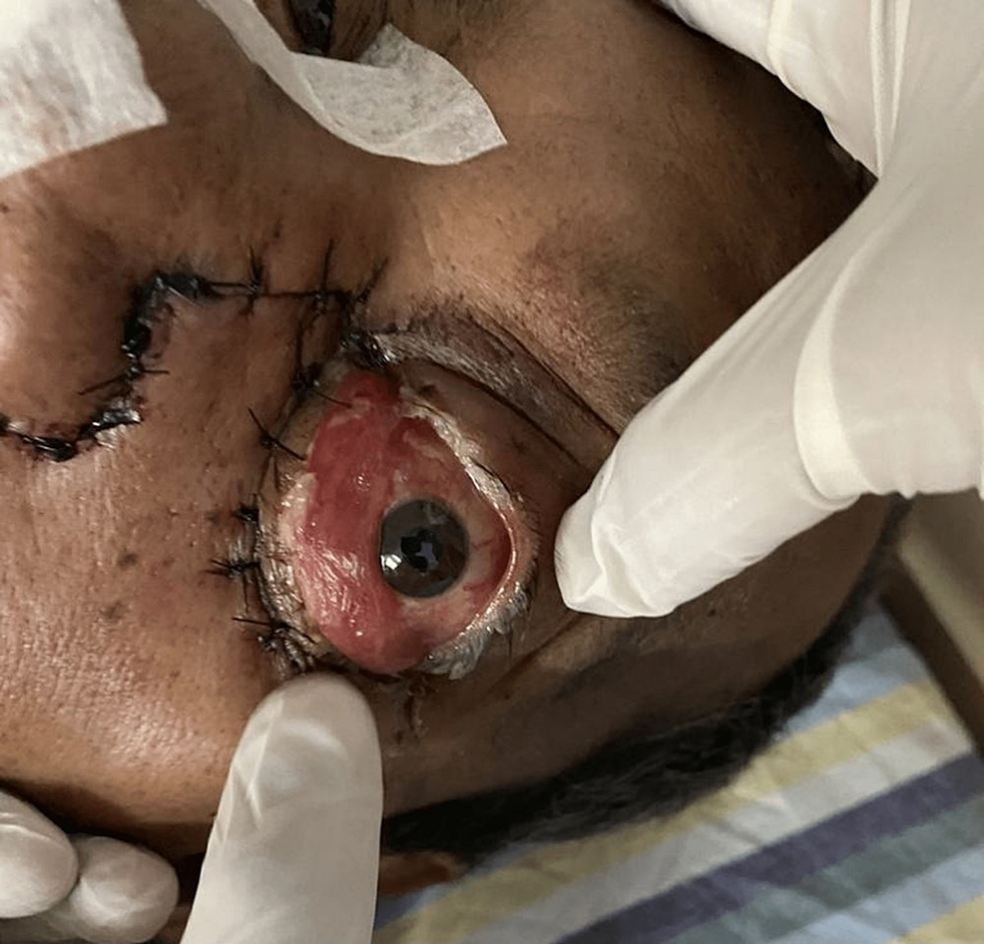
Patient with unilateral chemosis

**Figure 3:**
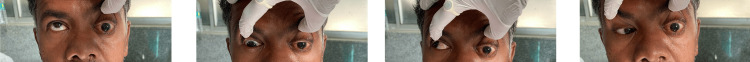
Patient showing complete unilateral ophthalmoplegia.

The most common ophthalmic clinical presentation (Table 4) of these patients was ptosis in 30 (75%) patients (Fig 1), 22 (55%) patients presented with proptosis (Fig 2), 19 patients (47.5%) had chemosis (Fig 2), 15 (37.5%) patients had limited ocular motility, 11 (27.5%) had complete ophthalmoplegia (Fig 3), and 11 (27.5%) patients had central retinal artery occlusion.

**Table 4:** Clinical features of patients diagnosed with rhino orbital mucormycosis

CLINICAL FEATURES	NUMBER OF PATIENTS (%)
Lid edema	33 (82.5)
Congestion	14 (35)
Chemosis	19 (47.5)
Limited ocular motility	15 (37.5)
Complete ophthalmoplegia	11 (27.5)
Ptosis	30 (75)
Proptosis	22 (55)
Central retinal artery occlusion	11 (27.5)

A total of 25 patients presented unilaterally (62.5%), and 15 (37.5%) patients had a bilateral presentation. Amongst the 25 unilateral orbital involvement cases, 10 showed a left-sided presentation, and 15 showed a right-sided presentation. All the patients presented with sinus involvement (100%). Maxillary sinus was most commonly affected (92.5%), followed by ethmoid sinus (87.59%), sphenoid sinus (75%), and frontal sinus (62.5%). CT Scan demonstrated diffuse mucosal thickening in paranasal sinuses. Magnetic resonance imaging with contrast was done for 10 patients suspecting intracranial spread. Three patients showed intracranial extension. One patient had focal edema in the basifrontal lobe, the second patient had changes suggestive of acute infarcts and abscesses, and the third patient had cavernous sinus thrombosis. Intravenous liposomal amphotericin B in a dosage of 3-5mg/kg body weight per day was given to all the patients included in our study. Nasal endoscopic debridement with orbital decompression was done in 22 patients (55%). 10 (25%) patients underwent only nasal endoscopic debridement, and five (12.5%) underwent maxillectomy. One patient underwent orbital exenteration with maxillectomy with endoscopic debridement, and specimens were sent for histopathology and microscopic examination. It revealed broad aseptate hyphae with right-angled branching.

## Discussion

Rhino-orbital-cerebral mucormycosis (ROCM) is a rare and life-threatening condition that is an invasive fungal infection that often occurs in individuals who are immunocompromised [7,8]. Mucormycosis is caused by fungi of the family mucoracea. The principal pathogens in this family are rhizopus, mucor, and absidia species. Mucoracea is found in soil, decaying vegetation, and other organic matter. Mucormycosis is a polymorphic disease with diverse clinical manifestations [9]. A complex reciprocation of factors, like diabetes mellitus, previous respiratory condition, use of immunosuppressive therapy, risk of hospital-acquired infections, and immune alterations systemically of COVID-19 infection may lead to secondary infections, which are increasingly being recognized due to their impact on morbidity and mortality [10]. There are specific pathophysiologic features of COVID-19 disease that may cause secondary fungal infections, causing extensive pulmonary disease and subsequent alveolar-interstitial pathology that may aggravate the risk of invasive fungal infections. The dysregulation associated with COVID-19, with the reduction of T lymphocytes, CD4+T, and CD8+T cells, may alter innate immunity [11]. It starts in the nose and paranasal sinuses but is often suspected following orbital spread, explaining its poor prognosis [12]. The infiltration of fungus destroys not only the surrounding bone but also the soft tissue through vascular thrombosis and subsequently can lead to tissue infarction and may reach the brain causing fatal complications [8].

The present study showed that Rhino orbital Mucormycosis was more prevalent in patients with COVID-19 infection (72.5%) and diabetes mellitus (75%), followed by the use of oxygen mask (62.5%) and steroids treatment (62.5%). The most common presentation in these patients included lid edema, congestion, chemosis, limited ocular motility, complete ophthalmoplegia, ptosis, proptosis, and central retinal artery occlusion.

The most common presentation in these patients included lid edema, congestion, chemosis, limited ocular motility, complete ophthalmoplegia, ptosis, proptosis, and central retinal artery occlusion, which were similar to the study done by Bayram et al., except endophthalmitis which was detected in 54.5% of patients. We did not find any endophthalmitis cases in our study [2]. The demographic profile of the patients in this study was similar to those observed in the literature [13].

In our study, the most commonly prevalent underlying risk factor was diabetes mellitus (75%). This could be due to the ketone reductase enzyme in the Mucorales, which thrive in hyperglycemia and diabetic ketoacidosis. In patients with COVID-19, a patient can develop diabetes mellitus or worsening hyperglycemia in already known diabetic patients due to damage to the b cells and reduced insulin secretion endogenously. Corticosteroids can further worsen glucose control and lead to immune system dysregulation, thereby predisposing to mucormycosis [14]. In these patients, corticosteroids were discontinued immediately, and either insulin therapy or oral hypoglycemic agents controlled blood sugars. 

Although there is no direct relationship between COVID-19 disease and Mucormycosis, there was a sudden surge in Mucormycosis cases during the second wave of the COVID-19 pandemic. This could be due to immune dysregulation with a reduction of several T lymphocytes, CD4+T, CD8+T cells, and higher proinflammatory cytokines like interleukin 1 (IL-1), interleukin 2 (IL-2), interleukin 6 (IL-6) and tumor necrosis factor α (TNF-α) [2]. In our study, 72.5% of patients had a present or history of COVID-19 infection, which was similar to the other study results [1,2]. However, 27.5% of patients were negative for COVID-19 infection, but 17.5% of patients were associated with diabetes mellitus, 7.5% were on corticosteroids, and 10% were negative for COVID-19 infection as well as diabetes mellitus. These suggest that other unknown mechanisms play a role in developing mucormycosis, which needs further studies. 

The other risk factors associated with mucormycosis were hematological malignancies, previous organ transplantation, immunosuppressive therapy, desferoxamine therapy, and patients on hemodialysis [13,15-18]. Our study did not find an association with any of these risk factors. A well-planned and early diagnosis with a multidisciplinary approach can help save life and sight of the patients. Over the years, microbiological diagnosis, controlling associated systemic conditions, medical treatment, and surgical debridement of necrotic tissue have been the mainstay of treatment of Mucormycosis. 

All the patients in our study received systemic liposomal amphotericin B, the first-line drug in the treatment of mucormycosis. It is less toxic and more effective without side effects. Due to vascular thrombosis and necrosis of tissues, amphotericin B may not reach an adequate concentration. Therefore, necrotic and infective tissue debridement is paramount for the antifungals to be effective.

The fungal infection can spread intracranially through the orbital apex, increasing morbidity and mortality. In our study, 7.5% of patients had intracranial involvement, and one underwent exenteration. There needs to be a clear-cut consensus on when exenteration should be performed. A study done by Hargrove et al. with 292 cases of orbital mucormycosis reported that there is no standard consensus on when exenteration has to be performed. Early diagnosis and immediate treatment with systemic and retrobulbar amphotericin B injections can prevent patients from undergoing exenteration [19].

Limitations

The limitations of our study were the small sample size and follow-up was not done for these patients.

## Conclusions

Rhino orbital Mucormycosis is more prevalent in patients with COVID-19 infection, primarily those who have used steroids and oxygen and with type 2 diabetes mellitus. An aggressive multidisciplinary approach is required with a high index of suspicion. Early diagnosis and prompt treatment with amphotericin B and surgical debridement can decrease the severity of rhino orbital Mucormycosis.
